# Supplementary material for: Downscaling satellite soil moisture using geomorphometry and machine learning
Source: PLoS One. 2019 Sep 24;14(9):e0219639. doi: 10.1371/journal.pone.0219639 (PMC6759172; doi:10.1371/journal.pone.0219639)
Supplement: S1 Appendix — (HTML) [file pone.0219639.s001.html]

Principal Component Analysis


# Principal Component Analysis

#### *Dataset dat*

This dataset contains 8108 individuals and 16 variables, 1 quantitative variable is considered as illustrative.

---

### 1. Study of the outliers

The analysis of the graphs leads to detect outliers that strongly influence the results. First we will describe these outliers and then we will suppress them from the analysis. Looking at the graph, we can note that 3 particular individuals strongly contribute to the construction of the plane. The cumulative contribution of these individuals to the construction of the plane equals **82.2%**.

**Figure 1.1 - Individuals factor map (PCA) before correction.** *Highlighting of 3 outliers.*

**Figure 1.2 - Individuals factor map (PCA) after correction.** *Highlighting of 3 outliers.*

**Figure 1.3 - Variables factor map (PCA) before correction** *The labeled variables are those the best shown on the plane.*

**Figure 1.3 - Variables factor map (PCA) after correction**

---

**The individual 5162** :

- takes very high values for the variable(s) :
- takes very low values for the variable(s) : *Relative.Slope.Position*, *Valley.Depth*, *Topographic.Wetness.Index* and *LS.Factor* (variables are sorted from the strongest).

**The individual 5267** :

- takes very high values for the variable(s) :
- takes very low values for the variable(s) : *Slope*, *Analytical.Hillshading*, *Catchment.Area*, *Convergence.Index*, *Vertical.Distance.to.Channel.Network*, *Channel.Network.Base.Level*, *Elevation*, *Relative.Slope.Position*, *Valley.Depth* and *Aspect* (variables are sorted from the strongest).

**The individual 6377** :

- takes very high values for the variable(s) :
- takes very low values for the variable(s) : *Slope*, *Analytical.Hillshading*, *Catchment.Area*, *Convergence.Index*, *Vertical.Distance.to.Channel.Network*, *Channel.Network.Base.Level*, *Elevation*, *Relative.Slope.Position*, *Valley.Depth* and *Aspect* (variables are sorted from the strongest).

These outliers are suppressed from the analysis and a second one is performed on the rest of the individuals.

---

### 2. Inertia distribution

The inertia of the first dimensions shows if there are strong relationships between variables and suggests the number of dimensions that should be studied.

The first two dimensions of PCA express **32.67%** of the total dataset inertia ; that means that 32.67% of the individuals (or variables) cloud total variability is explained by the plane. This is an intermediate percentage and the first plane represents a part of the data variability. This value is greater than the reference value that equals **14.34%**, the variability explained by this plane is thus significant (the reference value is the 0.95-quantile of the inertia percentages distribution obtained by simulating 547 data tables of equivalent size on the basis of a normal distribution).

From these observations, it may be interesting to consider the next dimensions which also express a high percentage of the total inertia.

**Figure 2 - Decomposition of the total inertia on the components of the PCA**

We can observe that the first 5 axis present an amount of inertia greater than those obtained by the 0.95-quantile of random distributions (68.22% against 35.11%). Thus, a wise decision would be to restrict the description to these only axis. However, we choosed to describe the first 8 axis.

---

### 3. Description of the plane 1:2

**Figure 3.1 - Individuals factor map (PCA)** *The labeled individuals are those with the higher contribution to the plane construction.*

**Figure 3.2 - Variables factor map (PCA)** *The variables in black are considered as active whereas those in blue are illustrative.* *The labeled variables are those the best shown on the plane.*

---

The **dimension 1** opposes individuals characterized by a strongly positive coordinate on the axis (to the right of the graph) to individuals characterized by a strongly negative coordinate on the axis (to the left of the graph).

The group 1 (characterized by a positive coordinate on the axis) is sharing :

- high values for the variables *Elevation*, *Channel.Network.Base.Level*, *Vertical.Distance.to.Channel.Network*, *Relative.Slope.Position*, *Cross.Sectional.Curvature*, *Convergence.Index*, *Longitudinal.Curvature* and *Slope* (variables are sorted from the strongest).
- low values for the variables *Valley.Depth*, *meanSM*, *Topographic.Wetness.Index*, *Closed.Depressions*, *LS.Factor*, *Catchment.Area* and *Analytical.Hillshading* (variables are sorted from the weakest).

The group 2 (characterized by a negative coordinate on the axis) is sharing :

- high values for the variables *Closed.Depressions*, *Topographic.Wetness.Index*, *Catchment.Area*, *Aspect*, *Channel.Network.Base.Level* and *LS.Factor* (variables are sorted from the strongest).
- low values for the variables *Convergence.Index*, *Slope*, *Longitudinal.Curvature*, *Cross.Sectional.Curvature*, *Analytical.Hillshading*, *Vertical.Distance.to.Channel.Network*, *Relative.Slope.Position* and *meanSM* (variables are sorted from the weakest).

The group 3 (characterized by a negative coordinate on the axis) is sharing :

- high values for the variables *Valley.Depth*, *meanSM*, *Analytical.Hillshading*, *Slope*, *Topographic.Wetness.Index*, *LS.Factor* and *Convergence.Index* (variables are sorted from the strongest).
- low values for the variables *Channel.Network.Base.Level*, *Elevation*, *Vertical.Distance.to.Channel.Network*, *Relative.Slope.Position*, *Closed.Depressions*, *Aspect*, *Cross.Sectional.Curvature* and *Catchment.Area* (variables are sorted from the weakest).

---

The **dimension 2** opposes individuals characterized by a strongly positive coordinate on the axis (to the top of the graph) to individuals characterized by a strongly negative coordinate on the axis (to the bottom of the graph).

The group 1 (characterized by a positive coordinate on the axis) is sharing :

- high values for the variables *Valley.Depth*, *meanSM*, *Analytical.Hillshading*, *Slope*, *Topographic.Wetness.Index*, *LS.Factor* and *Convergence.Index* (variables are sorted from the strongest).
- low values for the variables *Channel.Network.Base.Level*, *Elevation*, *Vertical.Distance.to.Channel.Network*, *Relative.Slope.Position*, *Closed.Depressions*, *Aspect*, *Cross.Sectional.Curvature* and *Catchment.Area* (variables are sorted from the weakest).

The group 2 (characterized by a negative coordinate on the axis) is sharing :

- high values for the variables *Closed.Depressions*, *Topographic.Wetness.Index*, *Catchment.Area*, *Aspect*, *Channel.Network.Base.Level* and *LS.Factor* (variables are sorted from the strongest).
- low values for the variables *Convergence.Index*, *Slope*, *Longitudinal.Curvature*, *Cross.Sectional.Curvature*, *Analytical.Hillshading*, *Vertical.Distance.to.Channel.Network*, *Relative.Slope.Position* and *meanSM* (variables are sorted from the weakest).

The group 3 (characterized by a negative coordinate on the axis) is sharing :

- high values for the variables *Elevation*, *Channel.Network.Base.Level*, *Vertical.Distance.to.Channel.Network*, *Relative.Slope.Position*, *Cross.Sectional.Curvature*, *Convergence.Index*, *Longitudinal.Curvature* and *Slope* (variables are sorted from the strongest).
- low values for the variables *Valley.Depth*, *meanSM*, *Topographic.Wetness.Index*, *Closed.Depressions*, *LS.Factor*, *Catchment.Area* and *Analytical.Hillshading* (variables are sorted from the weakest).

---

### 4. Description of the plane 3:4

**Figure 4.1 - Individuals factor map (PCA)** *The labeled individuals are those with the higher contribution to the plane construction.*

**Figure 4.2 - Variables factor map (PCA)** *The variables in black are considered as active whereas those in blue are illustrative.* *The labeled variables are those the best shown on the plane.*

---

The **dimension 3** opposes individuals characterized by a strongly positive coordinate on the axis (to the right of the graph) to individuals characterized by a strongly negative coordinate on the axis (to the left of the graph).

The group 1 (characterized by a positive coordinate on the axis) is sharing :

- high values for the variables *Channel.Network.Base.Level*, *Elevation*, *Closed.Depressions*, *Vertical.Distance.to.Channel.Network*, *Aspect*, *Catchment.Area*, *Cross.Sectional.Curvature*, *Topographic.Wetness.Index* and *LS.Factor* (variables are sorted from the strongest).
- low values for the variables *Analytical.Hillshading*, *Slope*, *meanSM*, *Valley.Depth*, *Longitudinal.Curvature* and *Convergence.Index* (variables are sorted from the weakest).

The group 2 (characterized by a positive coordinate on the axis) is sharing :

- high values for the variables *Analytical.Hillshading*, *Slope*, *meanSM*, *Valley.Depth*, *Longitudinal.Curvature*, *LS.Factor*, *Topographic.Wetness.Index*, *Convergence.Index* and *Relative.Slope.Position* (variables are sorted from the strongest).
- low values for the variables *Channel.Network.Base.Level*, *Elevation*, *Closed.Depressions*, *Aspect*, *Vertical.Distance.to.Channel.Network*, *Catchment.Area* and *Cross.Sectional.Curvature* (variables are sorted from the weakest).

The group 3 (characterized by a negative coordinate on the axis) is sharing :

- low values for the variables *Topographic.Wetness.Index*, *LS.Factor*, *Longitudinal.Curvature*, *Vertical.Distance.to.Channel.Network* and *Convergence.Index* (variables are sorted from the weakest).

The group 4 (characterized by a negative coordinate on the axis) is sharing :

- high values for the variable *Catchment.Area*.
- low values for the variables *Relative.Slope.Position* and *Valley.Depth* (variables are sorted from the weakest).

Note that the variables *Topographic.Wetness.Index* and *LS.Factor* are highly correlated with this dimension (respective correlation of 0.01, 0.01). These variables could therefore summarize themselve the dimension 3.

---

The **dimension 4** opposes individuals characterized by a strongly positive coordinate on the axis (to the top of the graph) to individuals characterized by a strongly negative coordinate on the axis (to the bottom of the graph).

The group 1 (characterized by a positive coordinate on the axis) is sharing :

- low values for the variables *Topographic.Wetness.Index*, *LS.Factor*, *Longitudinal.Curvature*, *Vertical.Distance.to.Channel.Network* and *Convergence.Index* (variables are sorted from the weakest).

The group 2 (characterized by a positive coordinate on the axis) is sharing :

- high values for the variables *Channel.Network.Base.Level*, *Elevation*, *Closed.Depressions*, *Vertical.Distance.to.Channel.Network*, *Aspect*, *Catchment.Area*, *Cross.Sectional.Curvature*, *Topographic.Wetness.Index* and *LS.Factor* (variables are sorted from the strongest).
- low values for the variables *Analytical.Hillshading*, *Slope*, *meanSM*, *Valley.Depth*, *Longitudinal.Curvature* and *Convergence.Index* (variables are sorted from the weakest).

The group 3 (characterized by a negative coordinate on the axis) is sharing :

- high values for the variable *Catchment.Area*.
- low values for the variables *Relative.Slope.Position* and *Valley.Depth* (variables are sorted from the weakest).

The group 4 (characterized by a negative coordinate on the axis) is sharing :

- high values for the variables *Analytical.Hillshading*, *Slope*, *meanSM*, *Valley.Depth*, *Longitudinal.Curvature*, *LS.Factor*, *Topographic.Wetness.Index*, *Convergence.Index* and *Relative.Slope.Position* (variables are sorted from the strongest).
- low values for the variables *Channel.Network.Base.Level*, *Elevation*, *Closed.Depressions*, *Aspect*, *Vertical.Distance.to.Channel.Network*, *Catchment.Area* and *Cross.Sectional.Curvature* (variables are sorted from the weakest).

---

### 5. Description of the plane 5:6

**Figure 5.1 - Individuals factor map (PCA)** *The labeled individuals are those with the higher contribution to the plane construction.*

**Figure 5.2 - Variables factor map (PCA)** *The variables in black are considered as active whereas those in blue are illustrative.* *The labeled variables are those the best shown on the plane.*

---

The **dimension 5** opposes individuals characterized by a strongly positive coordinate on the axis (to the right of the graph) to individuals characterized by a strongly negative coordinate on the axis (to the left of the graph).

The group 1 (characterized by a positive coordinate on the axis) is sharing :

- high values for the variables *Analytical.Hillshading*, *Slope*, *Elevation*, *Vertical.Distance.to.Channel.Network*, *Relative.Slope.Position*, *Channel.Network.Base.Level* and *Aspect* (variables are sorted from the strongest).
- low values for the variables *Closed.Depressions*, *Topographic.Wetness.Index*, *Cross.Sectional.Curvature*, *meanSM*, *Convergence.Index*, *Longitudinal.Curvature*, *Valley.Depth*, *Catchment.Area* and *LS.Factor* (variables are sorted from the weakest).

The group 2 (characterized by a negative coordinate on the axis) is sharing :

- high values for the variables *Topographic.Wetness.Index* and *Closed.Depressions* (variables are sorted from the strongest).
- low values for the variables *Aspect*, *Slope*, *Analytical.Hillshading*, *LS.Factor*, *Vertical.Distance.to.Channel.Network* and *Relative.Slope.Position* (variables are sorted from the weakest).

The group 3 (characterized by a negative coordinate on the axis) is sharing :

- high values for the variables *Catchment.Area*, *LS.Factor*, *Topographic.Wetness.Index*, *Closed.Depressions*, *Valley.Depth* and *meanSM* (variables are sorted from the strongest).
- low values for the variables *Elevation*, *Channel.Network.Base.Level*, *Vertical.Distance.to.Channel.Network*, *Relative.Slope.Position* and *Convergence.Index* (variables are sorted from the weakest).

The group 4 (characterized by a negative coordinate on the axis) is sharing :

- high values for the variables *Closed.Depressions*, *Cross.Sectional.Curvature*, *Topographic.Wetness.Index*, *Convergence.Index*, *Longitudinal.Curvature*, *meanSM*, *Valley.Depth*, *LS.Factor* and *Aspect* (variables are sorted from the strongest).
- low values for the variables *Analytical.Hillshading*, *Slope*, *Elevation*, *Vertical.Distance.to.Channel.Network*, *Relative.Slope.Position* and *Channel.Network.Base.Level* (variables are sorted from the weakest).

---

The **dimension 6** opposes individuals characterized by a strongly positive coordinate on the axis (to the top of the graph) to individuals characterized by a strongly negative coordinate on the axis (to the bottom of the graph).

The group 1 (characterized by a positive coordinate on the axis) is sharing :

- high values for the variables *Catchment.Area*, *LS.Factor*, *Topographic.Wetness.Index*, *Closed.Depressions*, *Valley.Depth* and *meanSM* (variables are sorted from the strongest).
- low values for the variables *Elevation*, *Channel.Network.Base.Level*, *Vertical.Distance.to.Channel.Network*, *Relative.Slope.Position* and *Convergence.Index* (variables are sorted from the weakest).

The group 2 (characterized by a negative coordinate on the axis) is sharing :

- high values for the variables *Topographic.Wetness.Index* and *Closed.Depressions* (variables are sorted from the strongest).
- low values for the variables *Aspect*, *Slope*, *Analytical.Hillshading*, *LS.Factor*, *Vertical.Distance.to.Channel.Network* and *Relative.Slope.Position* (variables are sorted from the weakest).

The group 3 (characterized by a negative coordinate on the axis) is sharing :

- high values for the variables *Closed.Depressions*, *Cross.Sectional.Curvature*, *Topographic.Wetness.Index*, *Convergence.Index*, *Longitudinal.Curvature*, *meanSM*, *Valley.Depth*, *LS.Factor* and *Aspect* (variables are sorted from the strongest).
- low values for the variables *Analytical.Hillshading*, *Slope*, *Elevation*, *Vertical.Distance.to.Channel.Network*, *Relative.Slope.Position* and *Channel.Network.Base.Level* (variables are sorted from the weakest).

The group 4 (characterized by a negative coordinate on the axis) is sharing :

- high values for the variables *Analytical.Hillshading*, *Slope*, *Elevation*, *Vertical.Distance.to.Channel.Network*, *Relative.Slope.Position*, *Channel.Network.Base.Level* and *Aspect* (variables are sorted from the strongest).
- low values for the variables *Closed.Depressions*, *Topographic.Wetness.Index*, *Cross.Sectional.Curvature*, *meanSM*, *Convergence.Index*, *Longitudinal.Curvature*, *Valley.Depth*, *Catchment.Area* and *LS.Factor* (variables are sorted from the weakest).

---

### 6. Description of the plane 7:8

**Figure 6.1 - Individuals factor map (PCA)** *The labeled individuals are those with the higher contribution to the plane construction.*

**Figure 6.2 - Variables factor map (PCA)** *The variables in black are considered as active whereas those in blue are illustrative.* *The labeled variables are those the best shown on the plane.*

---

The **dimension 7** opposes individuals characterized by a strongly positive coordinate on the axis (to the right of the graph) to individuals characterized by a strongly negative coordinate on the axis (to the left of the graph).

The group 1 (characterized by a positive coordinate on the axis) is sharing :

- high values for the variables *Analytical.Hillshading*, *Closed.Depressions*, *Channel.Network.Base.Level*, *Elevation*, *Topographic.Wetness.Index*, *LS.Factor*, *Convergence.Index*, *Cross.Sectional.Curvature*, *Slope* and *Aspect* (variables are sorted from the strongest).
- low values for the variables *meanSM*, *Valley.Depth*, *Vertical.Distance.to.Channel.Network*, *Longitudinal.Curvature*, *Catchment.Area* and *Relative.Slope.Position* (variables are sorted from the weakest).

The group 2 (characterized by a positive coordinate on the axis) is sharing :

- high values for the variables *Topographic.Wetness.Index*, *Closed.Depressions*, *Channel.Network.Base.Level* and *Cross.Sectional.Curvature* (variables are sorted from the strongest).
- low values for the variables *Aspect*, *Slope*, *LS.Factor*, *Analytical.Hillshading*, *Relative.Slope.Position* and *Vertical.Distance.to.Channel.Network* (variables are sorted from the weakest).

The group 3 (characterized by a negative coordinate on the axis) is sharing :

- high values for the variables *meanSM*, *Valley.Depth*, *Vertical.Distance.to.Channel.Network*, *Longitudinal.Curvature*, *Aspect*, *Catchment.Area*, *Relative.Slope.Position* and *Slope* (variables are sorted from the strongest).
- low values for the variables *Analytical.Hillshading*, *Closed.Depressions*, *Channel.Network.Base.Level*, *Topographic.Wetness.Index*, *Elevation*, *Convergence.Index*, *LS.Factor* and *Cross.Sectional.Curvature* (variables are sorted from the weakest).

---

The **dimension 8** opposes individuals characterized by a strongly positive coordinate on the axis (to the top of the graph) to individuals characterized by a strongly negative coordinate on the axis (to the bottom of the graph).

The group 1 (characterized by a positive coordinate on the axis) is sharing :

- high values for the variables *Analytical.Hillshading*, *Closed.Depressions*, *Channel.Network.Base.Level*, *Elevation*, *Topographic.Wetness.Index*, *LS.Factor*, *Convergence.Index*, *Cross.Sectional.Curvature*, *Slope* and *Aspect* (variables are sorted from the strongest).
- low values for the variables *meanSM*, *Valley.Depth*, *Vertical.Distance.to.Channel.Network*, *Longitudinal.Curvature*, *Catchment.Area* and *Relative.Slope.Position* (variables are sorted from the weakest).

The group 2 (characterized by a negative coordinate on the axis) is sharing :

- high values for the variables *meanSM*, *Valley.Depth*, *Vertical.Distance.to.Channel.Network*, *Longitudinal.Curvature*, *Aspect*, *Catchment.Area*, *Relative.Slope.Position* and *Slope* (variables are sorted from the strongest).
- low values for the variables *Analytical.Hillshading*, *Closed.Depressions*, *Channel.Network.Base.Level*, *Topographic.Wetness.Index*, *Elevation*, *Convergence.Index*, *LS.Factor* and *Cross.Sectional.Curvature* (variables are sorted from the weakest).

The group 3 (characterized by a negative coordinate on the axis) is sharing :

- high values for the variables *Topographic.Wetness.Index*, *Closed.Depressions*, *Channel.Network.Base.Level* and *Cross.Sectional.Curvature* (variables are sorted from the strongest).
- low values for the variables *Aspect*, *Slope*, *LS.Factor*, *Analytical.Hillshading*, *Relative.Slope.Position* and *Vertical.Distance.to.Channel.Network* (variables are sorted from the weakest).

---

### 7. Classification

The dataset is too large to perform the classification.

---

## Annexes
