# Supplementary figures and images for: Downscaling satellite soil moisture using geomorphometry and machine learning
Source: PLoS One. 2019 Sep 24;14(9):e0219639. doi: 10.1371/journal.pone.0219639 (PMC6759172; doi:10.1371/journal.pone.0219639)

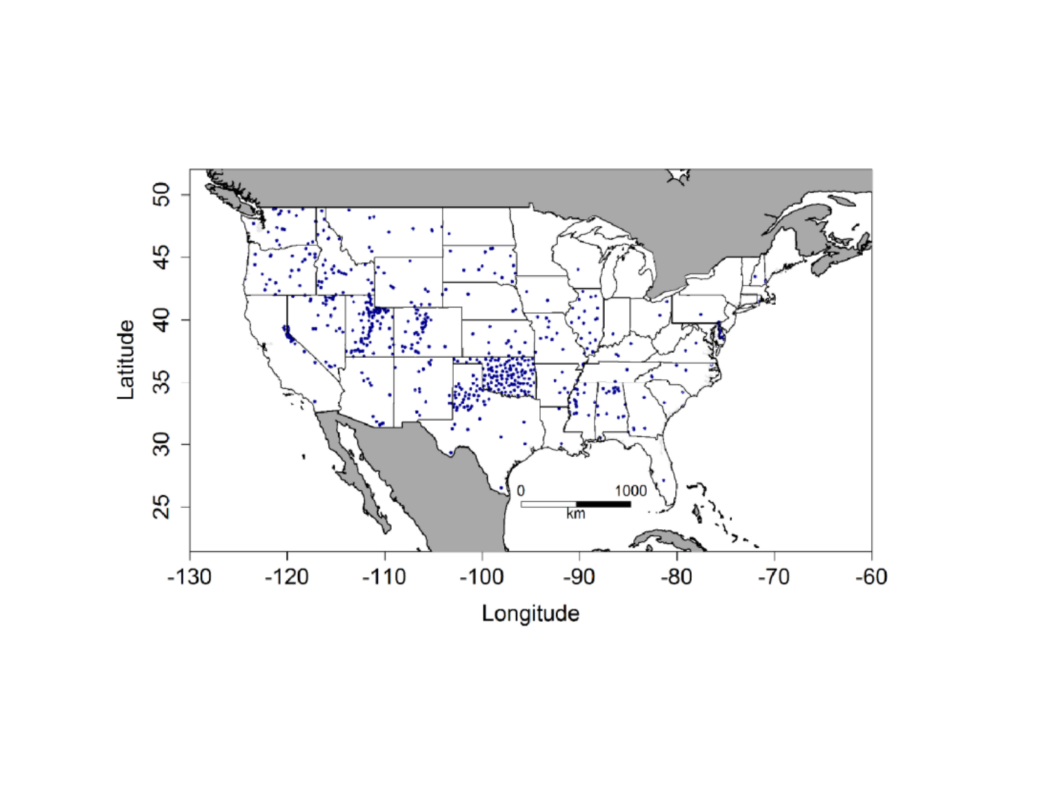

Supplement: S1 Fig — (TIF) [file pone.0219639.s002.tif]

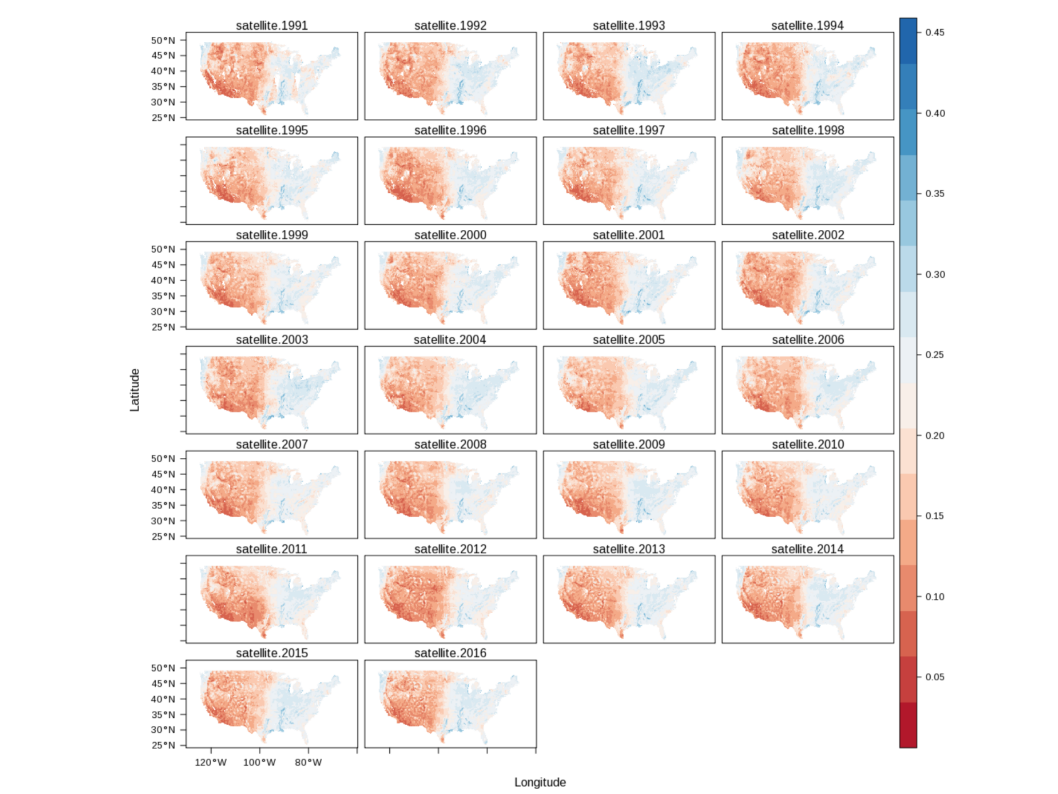

Supplement: S2 Fig — (TIF) [file pone.0219639.s003.tif]

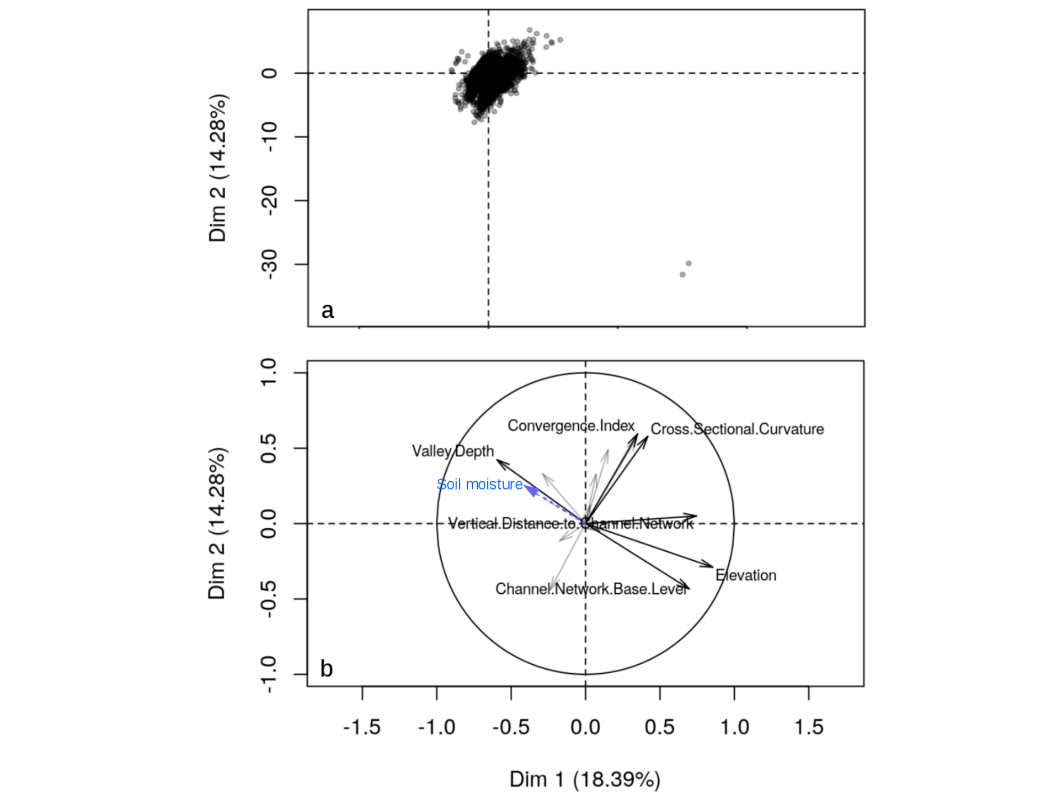

Supplement: S3 Fig — The individual point cloud across the plane between the first and second PCA (a). The orthogonal relationship of the variables with higher contribution to this plane (b). Soil moisture is represented by the dotted blue line. An interpretation of these can be found in S1 Appendix. (TIF) [file pone.0219639.s004.tif]

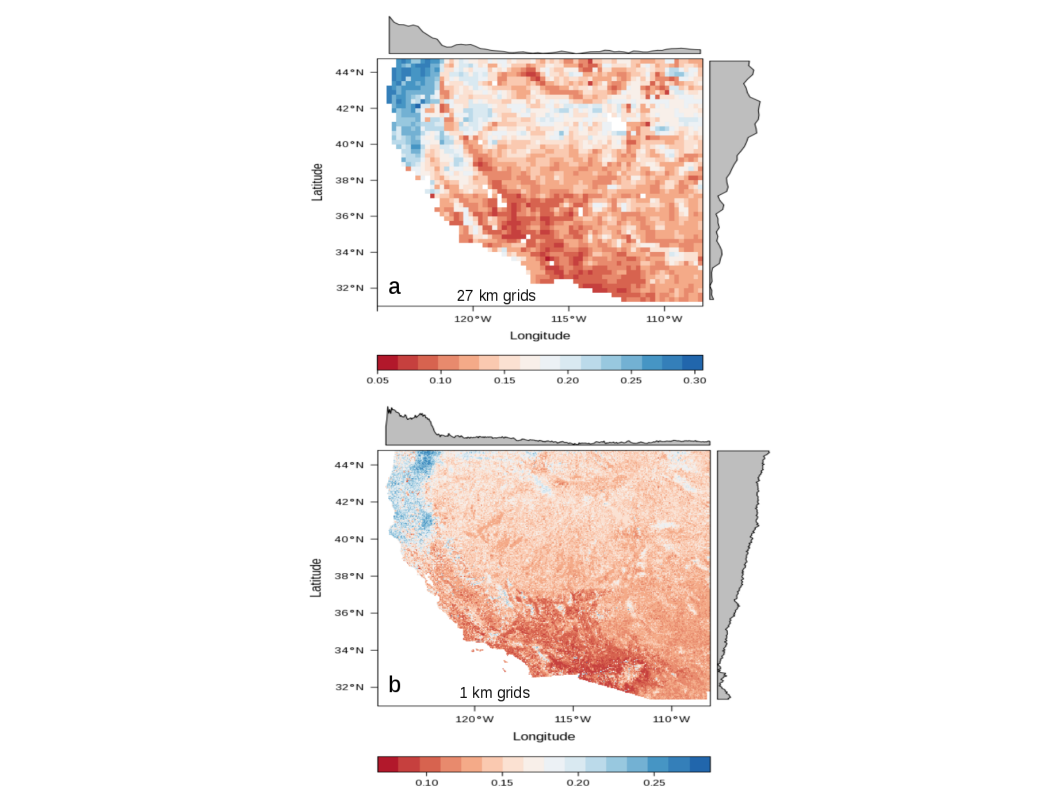

Supplement: S4 Fig — Comparison between the original satellite soil moisture (a) and soil moisture predicted at 1km grids (b). (TIF) [file pone.0219639.s005.tif]

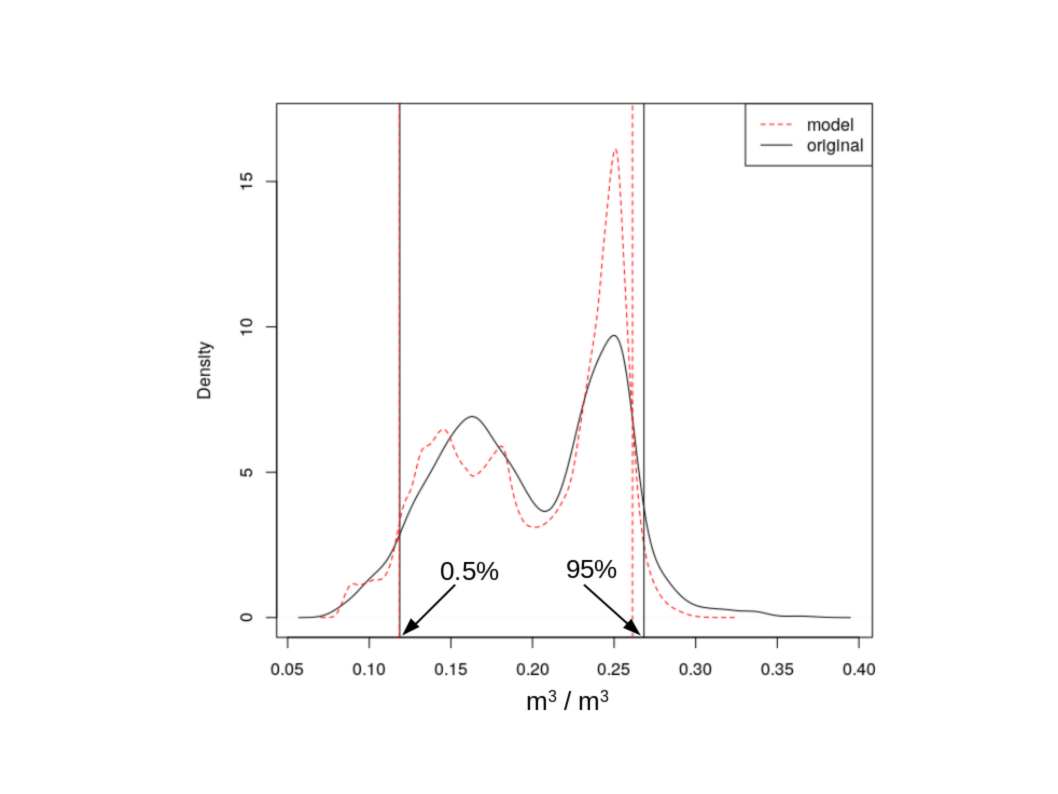

Supplement: S5 Fig — Bimodal distribution of satellite soil moisture (black) and the downscaled soil moisture estimates (red). (TIF) [file pone.0219639.s006.tif]
