# Supplementary material for: Downscaling satellite soil moisture using geomorphometry and machine learning
Source: PLoS One. 2019 Sep 24;14(9):e0219639. doi: 10.1371/journal.pone.0219639 (PMC6759172; doi:10.1371/journal.pone.0219639)
Supplement: S1 Table — (DOCX) [file pone.0219639.s007.docx]

Table S1. Terrain parameters generated across 1km grids using digital terrain analysis. These terrain parameters were used as prediction factors for soil moisture across CONUS. They were selected given its high influence on soil moisture variability and other soil assessments as documented in previous studies.

| CODE | VARIABLE | DESCRIPTION | UNITS |
| --- | --- | --- | --- |
| aspect | Terrain aspect | The orientation of the terrain slope | Radians |
| carea | Catchment area | Specific catchment area, the area from which rainfall flows into a river, lake, or reservoir. | Square meters |
| chnl_base | Channel network base level | Chanel network base level, the distance of each pixel to highest surrounding elevation | Meters |
| chnl_dist | Distance to channel network | Distance from each pixel to the nearest channel network, stream or river | Meters |
| convergence | Convergence index | Convergence index, This index is obtained by averaging the bias of the slope directions of the adjacent cells from the direction of the central cell, and subtracting 90 degrees. | The possible values of the index range from -90° to +90° |
| hcurv | Horizontal curvature | Plan curvature, the curvature of the hillside in a horizontal plane. Plan curvature is the horizontal curvature, intersecting with the XY plane. | This index is centered in zero values and indicates the concavity or convexity of the terrain surface |
| land | DEM, elevation | Digital elevation model 1x1km | Meters above sea level |
| lsfactor | Length slope factor | Length of constant slopes from-to a given point | Meters |
| rsp | Relative slope position | The position of relative height maxima as a function of slope. | Continuous variable ranging from 0 (valley floor) to 100 (ridge top) |
| shade | Analytical hillshading | Analytical hillshading. The representation of topographic relief with the method of hill-shading, based on simulating the effect of natural light on earth's surface | Adim. Higher values are receiving more light given a specific position. |
| sinks | Filtered DEM | Elevation data after using a method for identifying and filling surface depressions in digital elevation models for hydrologic analysis and modelling | Meters above sea level |
| slope | Terrain slope | The change of elevation while increasing distance. Is the measure of steepness or the degree of inclination of a feature relative to the horizontal plane. | Radians |
| vall_depth | Valley depth index | Interpolation of a channel network base level elevation,  and the subtraction of this base level from the original elevations. | Meters. |
| vcurv | Vertical curvature | Profile curvature is the curvature intersecting with the plane defined by the Z axis and maximum gradient direction. Positive values describe convex profile curvature, negative values concave profile. | This index is centered in zero values and indicates the concavity or convexity of the terrain surface |
| wetness | Topographic wetness index | This index is indicator for the likelihood of saturated soil conditions during rain events and as an indicator for sediment and matter accumulation. In the absence of information on hydraulic soil transmissivity. | Adim. Higher values indicate areas where water tends to accumulate. |

References:

Boehner, J. and Selige, T. (2006): Spatial prediction of soil attributes using terrain analysis and climate regionalisation. In: Boehner, J., McCloy, K.R., Strobl, J. [Ed.]: SAGA - Analysis and Modelling Applications, Goettinger Geographische Abhandlungen, Goettingen: 13-28.

Conrad, O., Bechtel, B., Bock, M., Dietrich, H., Fischer, E., Gerlitz, L., ...Böhner, J. (2015). System for Automated Geoscientific Analyses (SAGA) v. 2.1.4. Geosci. Model Dev. Discuss., 8, 2271–2312. doi: 10.5194/gmdd-8-2271-2015

Moore, I. D., Gessler, P. E., Nielsen, G. A., & Peterson, G. A. (1993). Soil Attribute Prediction Using Terrain Analysis. Soil Sci. Soc. Am. J., 57(2), NP–NP. doi: 10.2136/sssaj1993.03615995005700020058x

Vorpahl P.; Elsenbeer H.; Märker M.; Schröder B. How can statistical models help to determine driving factors of landslides? Ecol. Modell., vol. 239, 24 July 2012, pp. 27-39, doi:10.1016/j.ecolmodel.2011.12.007.

Wang, L. & H. Liu (2006): An efficient method for identifying and filling surface depressions in digital elevation models for hydrologic analysis and modelling. International Journal of Geographical Information Science, Vol. 20, No. 2: 193-213.

Wood, J. (1996): The Geomorphological characterisation of Digital Elevation Models. Diss., Department of Geography, University of Leicester, U.K. [online](http://www.soi.city.ac.uk/~jwo/phd/).

Wood, J. (2009): Geomorphometry in LandSerf. In: Hengl, T. and Reuter, H.I. [Eds.]: Geomorphometry: Concepts, Software, Applications. Developments in Soil Science, Elsevier, Vol.33, 333-349.
